# Computational annotation of UTR cis-regulatory modules through Frequent Pattern Mining

**DOI:** 10.1186/1471-2105-10-S6-S25

**Published:** 2009-06-16

**Authors:** Antonio Turi, Corrado Loglisci, Eliana Salvemini, Giorgio Grillo, Donato Malerba, Domenica D'Elia

**Affiliations:** 1 Department of Computer Science – University of Bari, Via Orabona 4, 70125 Bari, Italy; 2 Institute for Biomedical Technologies, CNR, Via Amendola 122/D, 70126 Bari, Italy

## Abstract

**Background:**

Many studies report about detection and functional characterization of cis-regulatory motifs in untranslated regions (UTRs) of mRNAs but little is known about the nature and functional role of their distribution. To address this issue we have developed a computational approach based on the use of data mining techniques. The idea is that of mining frequent combinations of translation regulatory motifs, since their significant co-occurrences could reveal functional relationships important for the post-transcriptional control of gene expression. The experimentation has been focused on targeted mitochondrial transcripts to elucidate the role of translational control in mitochondrial biogenesis and function.

**Results:**

The analysis is based on a two-stepped procedure using a sequential pattern mining algorithm. The first step searches for frequent patterns (FPs) of motifs without taking into account their spatial displacement. In the second step, frequent sequential patterns (FSPs) of spaced motifs are generated by taking into account the conservation of spacers between each ordered pair of co-occurring motifs. The algorithm makes no assumption on the relation among motifs and on the number of motifs involved in a pattern. Different FSPs can be found depending on different combinations of two parameters, i.e. the threshold of the minimum percentage of sequences supporting the pattern, and the granularity of spacer discretization. Results can be retrieved at the UTRminer web site: . The discovered FPs of motifs amount to 216 in the overall dataset and to 140 in the human subset. For each FP, the system provides information on the discovered FSPs, if any. A variety of search options help users in browsing the web resource. The list of sequence IDs supporting each pattern can be used for the retrieval of information from the UTRminer database.

**Conclusion:**

Computational prediction of structural properties of regulatory sequences is not trivial. The presented data mining approach is able to overcome some limits observed in other competitive tools. Preliminary results on UTR sequences from nuclear transcripts targeting mitochondria are promising and lead us to be confident on the effectiveness of the approach for future developments.

## Background

The huge amount of data recently produced by genome sequencing projects has allowed to highlight information on the genetic content of many organisms in the form of lists of all genes they can express. Although necessary, this knowledge is not sufficient to understand mechanisms ruling cell growth, differentiation, development and many other events underlying life. In this sense, it is crucial to decipher the control mechanisms that regulate the expression of genes in time and space. Regulated translation plays an important role in this control mainly by means of regulatory proteins that recognize functional motifs located in the untranslated region upstream (5'UTR) and downstream (3'UTR) of mRNA coding sequence [1]. Evidences from recent studies support the idea that in some cases, for a correct functional interaction, different features of both regulatory motifs and UTR sequence context are necessary [2]. The presence of multiple copies of the same motif along a UTR sequence as well as the combinatorial regulation by multiple classes of motifs may also be important. One of the most typical example is represented by the microRNAs (miRNA), small regulatory RNAs that control gene expression by binding to complementary sites in target mRNAs. A study on the specificity of miRNA target selection in translation repression has demonstrated that a mRNA can be simultaneously repressed by more than one miRNA species and that the level of repression achieved is dependent on both the amount of mRNA and the amount of available miRNA complexes. In addition, authors report that also distances among target sites are important for their activity [3].

These findings underline that regulated translation is much more complex than expected and that predicted interactions among translational control elements must be viewed in a broader context than that of the simple interaction between the regulatory factor and its target site. Spatial configuration of motifs along the UTR sequences may play an important role and should be considered for the understanding of their mechanism of action.

The problem of motif discovery has been widely investigated in literature and a lot of pattern discovery algorithms have been proposed [4]. HeliCis [5] is a motifs discovery tool based on a probabilistic model, which allows de novo discovery of motif pairs with periodic spacing of fixed length (helical phasing). The limits of this algorithm are the dimension of the pattern (dyad pattern) and its lack of flexibility in defining the length of the spacer between the two monad motifs. Differently from Helicis, BioProspector [6] can detect patterns formed by two motifs, m1 and m2, in which the gap varies in a range (g1, g2), but it needs to know, as input parameters, the width of the component motifs m1 and m2 and their gap range. MITRA [7] is another tool that finds only dyad patterns, but in this case the two monad motifs must be at a fixed range distance far each other. TEIRESIAS [8] is able to detect composite patterns with variable length, but the length of the gap needs to be set a priori and cannot vary in the different instances of the pattern, that is to say the length of the same gap is not flexible. Another tool facing the motif-finding problem is SPACE [9] whose strategy is similar to that of TEIRESIAS. It discovers patterns, which can be composed by many monad motifs interleaved by gaps whose length has not to be defined a priori, but it does not allow for varying the length of the same gap in the various instances of the same pattern.

The aim of the present work is the development of a bioinformatic tool providing annotations of both nature, distribution and frequent association of regulatory motifs in UTR sequences, that is to discover Frequent Sequential Pattern (FSPs) of spaced motifs. We have developed a two-stepped procedure making use of data mining techniques. Through this approach, we are able to map motifs along UTR sequences, to measure inter-motif distances and to evaluate FSPs involving several motifs, without any prior assumption on their distribution. In particular, the approach first finds out co-occurring sets of motifs without taking into account their spatial displacement, then generates FSPs of spaced motifs (namely, motifs separated by spacers of different lengths) from a specific set of motifs previously mapped.

Our tool is different from other competitive pattern discovery tools because it is able to investigate the exploration of patterns made by several motifs whose length is not predefined and whose spacers can have a variable width (within some interval). Here we present results obtained on UTR sequences extracted from nuclear mRNAs targeting mitochondria and discuss how these results may provide useful clues for experimental studies addressed to the elucidation of mechanisms regulating translation.

## Methods

### Generation of the UTRminer data collection and UTRminer database

**Figure 1:**
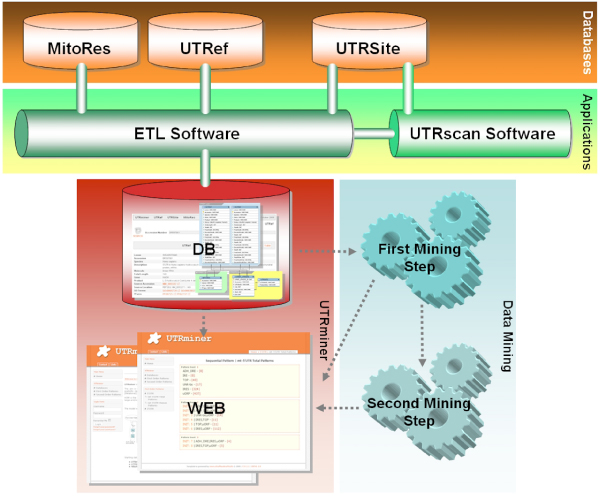
**Data resources used for the UTRminer generation and the processing flow**. The UTRminer database integrates data of MitoRes database with: i) UTR sequences collected from the UTRef database, ii) UTR regulatory motifs extracted from the UTRsite database, including standard name, description and pattern annotation syntax. Data on species, gene name, protein function, UTRef ID reference, sequence length and base composition are extracted from the UTRef database. Gene Ontology (GO) and PFam IDs are directly collected from MitoRes through the RefSeq collection.

Figure 1 presents a schema of the generation of the UTRminer resource. The UTRminer dataset was generated by using the gene collection of the MitoRes database [10] as a reference for mitochondrial genes, and by exploiting its cross-referencing with the UTRef database [11] for the downloading of the corresponding UTR sequences. Sequences extracted from UTRef database were annotated by using UTRscan, which looks for UTR functional elements by searching through submitted sequence data. The software UTRscan searches for motif patterns collected in the UTRsite database by using the PatSearch pattern syntax [12]. Analyzed sequences included the first and last 100 nt of the coding sequence (CDS) in the 5'- and 3'-UTRs respectively to allow the detection of UTR-CDS overlapping motifs. UTRsite motifs detected in each UTR sequence were annotated in terms of their start and end position along UTRs and distance from the "start" and "end" position of the CDS in 5'UTRs and 3'UTRs, respectively. A MySQL database, named UTRminer, has been developed to collect this data and support the computational annotation, analysis and validation of FSPs discovered. In order to build up and run the whole ETL (Extract, Transform, Load) process for the population and annotation of the database, we developed an application, which manages connections with the related databases and uses the UTRscan engine for the annotation of regulatory motifs.

### Generation of Frequent Sequential Patterns

The generation of the FSPs proceeds in two steps. In the first step, *sets *of motifs are found without taking into account the order in which motifs occur in the UTRs. These sets will be hereafter reported as frequent patterns (FP). In the second step, the focus is on the *sequences *of motifs (the FSPs) which also take into account the spatial displacement of motifs, that is, the spacers between motifs. *Sequences *are determined only for those sets which are deemed more interesting for some reasons, for instance, because their support is significantly high. The support of a sequential pattern is computed as the percentage of sequences in the dataset that contain the pattern in the same order.

The motivation for this double step is twofold: timing the computational complexity and making the biological analysis of patterns more manageable. As to the first motivation, we observe that algorithms for mining either frequent patterns or frequent sequential patterns are characterized by a very large computational complexity. In the case of frequent patterns, their enumeration takes place in the powerset 2^M ^where M is the set of motifs considered in the problem at hand. The case of sequential patterns is even more problematic, since the size of the search space is (2^M^)^p ^where *p *stands for the length of the longest sequence of motifs that can be discovered. The consideration of spacers adds further computational complexity. Therefore, the first step aims at identifying the sets of motifs, which should be considered in the sequence, thus reducing the size of M. The additional advantage is that the biological analysis can be focused on some motifs, making easier the interpretation of results.

Input data of the first mining step is a view on MitoRes, UTRef and UTRSite which associates UTRs to their contained motifs (see Figure 1). The search is based on the levelwise method by Mannila and Toivonen [13], i.e. a breadth-first search is performed in the lattice of sets of motifs. More precisely, the search starts from the smallest element (sets with a single motif) and proceeds from smaller to larger sets. The set of motifs which are frequent at the i-th level are considered to generate candidate sets of motifs at the (i+1)-th level. Candidates are then evaluated against the input data in order to prune those that are infrequent, i.e. those with a lower support than a defined threshold.

As a result of the first mining step, we have frequent associations of input motifs annotated with the first and the last nucleotide that localize the motif in the UTR sequence. Given two distinct annotated motifs p_1_^a ^and p_2_^a^, their placement in space is projected in a sequence of motifs ordered on the basis of the values of *startingPosition *(first nucleotide). The spacer between two consecutive motifs [*md(p1, p2)*] is computed as the difference between the *endingPosition *(last nucleotide) of p_1 _and the *startingPosition *of p_2_. Therefore, an UTR is modelled as a sequence of motifs with spacers in between. For instance, given two annotated motifs p_1_^a^: <p_1_, 100, 200> and p_2_^a^: < p_2_, 250, 300>, they generate the sequence < p_1_^a^, p_2_^a^> = < p_1_, 50, p_2_> since the *endingPosition *of p_1 _(200) precedes the *startingPosition *of p_2 _(250).

In the second step, FSPs are generated using the algorithm GSP (Generalized Sequential Pattern) [14], available in the Weka data mining tool [15]. GSP is based on the Apriori algorithm [16] like many other sequential pattern mining algorithms. The Apriori property proposed in association rule mining, states that any sub-pattern of a frequent pattern must be frequent. In this view, GSP tries to mine the most frequent association of spaced motif patterns considering a spacer or a motif simply as an item which can be frequent or not and, starting from frequent subset, it tries to generate bigger frequent sets which contain them.

In FSP, separating spacers can have different lengths for each ordered pair of motifs. The length of a spacer between two motifs is an integer number, negative or positive depending on motifs displacement. In order to allow GSP to extract FSPs, the length of spacers must be discretized. Discretization converts the original set of integer values into a set of discrete values, each of which is associated with an interval (or *bin*), which introduces a certain tolerance in the length of a spacer. By varying the number of *bins *in which all the spacers of a FSP can be discretized, it is possible to obtain predictions at different granularity levels. As an example, discretizing at 6 *bins *the set of spacers of a pattern, means to cluster them into 6 different size classes. If the same set of spacers is discretized at 9 *bins*, it means that 9 different size classes smaller than the one produced by the discretization at 6 *bins *will be considered. This makes prediction more stringent because it improves the sensitivity of the sequential pattern miner in selecting FSPs whose spacers are really very similar in size.

The discretization method used in this work is based on the principle of equal frequency for each interval (*bin*) according to which numerical values are approximately uniformly distributed among non-overlapping intervals of different width. An alternative to this method is the equal width discretization, which divides the range of numerical values into N intervals of equal width. However, equal width discretization is more sensitive to outliers and it does not handle well skewed data. On the contrary, equal frequency discretization is good for scaling data and it minimizes the information loss during the partitioning process. In the context of this work, equal frequency discretization presents two additional advantages: i) it is possible to discover motif patterns whose spacers are not forced to range in the same interval; ii) by varying the granularity of the discretization, it is possible to get information on the variability of the spacers over all the discovered patterns.

After spacer discretization, the representation of spaced motifs is changed. For example, if <A, 30, B, 1000, C, -200, D> is a sequence of spaced motifs, and {NEG DISTANCE, SHORT DISTANCE, LONG DISTANCE} are three discrete values corresponding to the intervals [-300, -1], [0, 210], [211, 1100], then the original sequence is transformed into the following one:

< A, SHORT DISTANCE, B, LONG DISTANCE, C, NEG DISTANCE, D >

This new representation of spaced motifs is considered when mining FSPs. A sequential pattern takes the form <motif_1, spacer, motif_2, spacer, . . ., motif_N-1, spacer, motif_N >: it begins and ends with a motif and has a support greater than an input threshold (*minsup*). Each sequence in the data set can count up to 1 in the computation of the support. Therefore the support is not affected by the length of the UTRs. The GSP jobs were executed over the EGEE [17] distributed grid infrastructure, which exploits the gLite middleware [18]. We subdivided the entire problem into elementary tasks, each one corresponding to the analysis of a single set of co-occurring motifs. The tasks were loaded into a DB server to create a "task queue" and jobs were executed using the Job Submission Tool (JST) [19]. The tool takes care of the submission to the grid, bookkeeping, resubmission in case of failures and monitoring of all the jobs necessary to complete the analysis with a very limited human intervention. For this application, particular care was taken to correctly recreate the environment in each work node and a procedure was set up to download and install the required Java package on the work node. The analysis script was then executed only if the installation of the Java package was successful. This strategy allowed us to increase the job success rate from 50% to 95%. For this run, we split the analysis in about 2000 different tasks for a total of about 300 CPU days. By using the EGEE infrastructure, the complete run was executed in about 2 days using, with a speedup factor of about 150.

## Results and discussion

UTR sequences have been divided into two main collections to evaluate the performance of the data mining software when analysing different type of samples. The first collection, named M_dataset, consists of 1944 5'UTR and 1952 3'UTR sequences from 10 different species. The second collection, named H_dataset, consists of 728 5'UTR and 728 3' UTR sequences from the human subset of the M_dataset collection. In the following paragraphs, we report and discuss: i) nature and distribution of translation regulatory motifs in the sample under investigation; ii) the results obtained on these annotations by the two-stepped data mining approach; iii) few examples of interesting structural features of UTR regulatory motifs detected through our analysis; iv) a general description of the UTRminer web site.

### UTR regulatory motif annotation: an overview of the mitochondrial dataset

Mapping regulatory motifs along the UTR sequences under investigation was done by searching for 46 motif patterns collected in the UTRsite database. Table 1 reports the number of the analyzed 5'- and 3'-UTR sequences, and the corresponding number of sequences bearing at least one regulatory motif occurrence. These sequences are those subsequently submitted to the data mining process for the discovery of FSPs. In this table we also report the lowest (min) and highest (max) number of target site occurrences detected along the same UTR sequence including multiple occurrences of the same motifs. The number of sequences bearing at least one target site occurrence is very low in 5' UTR collections from all examined species, while it ranges from 88% to 97% in 3' UTRs. As for the number of regulatory target sites present in these regions, we observe again a great difference when comparing the 5'-with the 3'-UTR collections. In 5' UTRs it ranges from 1 to 10 occurrences, and in 3' UTRs from 1 to 66.

**Table 1:** Statistics of UTR sequences collected in UTRminer.

	***5'UTR***				***3'UTR***			
	
	*A*	*B*	*C*	*D*	*E*	*F*	*G*	*H*
*Species*			*min*	*max*			*min*	*max*

*H. sapiens*	728	299	1	10	728	707	1	66
*M. musculus*	504	164	1	6	505	491	1	39
*D. melanogaster*	242	116	1	5	243	239	1	11
*R. norvegicus*	199	49	1	3	202	194	1	39
*B. taurus*	121	18	1	5	121	111	1	19
*D. rerio*	73	32	1	3	73	70	1	29
*S. scrofa*	28	3	1	1	29	28	1	20
*C. elegans*	24	2	2	3	23	21	1	6
*G. gallus*	16	6	1	3	19	18	1	9
*X. tropicalis*	9	1	1	1	9	8	1	8
	*1944*	*690*			*1952*	*1888*		

The table reports, for the 5'- and 3'-UTR datasets of each species: i) the number of pre-processed sequences with the UTRscan software (columns A and E); the number of sequences bringing at least one regulatory RNA motif (columns B and F); the lowest (columns C and G) and highest (columns D and H) number of cis-regulatory motif occurrences found along a same UTR sequence.

By pushing forward the original design goals of the UTRef database, we searched for Internal Ribosome Entry Site (IRES) also in the 3' UTR sequences due to findings on mitochondrial differentiation in which an IRES-like activity and sequence elements responsible for such activity, have been detected in the 3' UTR sequence of key respiratory chain mRNAs [20-23]. The same was done for the upstream Open Reading Frame (uORF) to investigate their potential role also in 3' UTRs. Indeed, the specific role of uORF is particularly intriguing due to the complexity of the mechanisms through which they are able to modulate the expression of mRNAs, and many recent works suggest a more complex implication of these elements in regulated translation [24].

The motifs frequently found in our sample are 18 and a list is provided in table 2. An interesting feature underlined by these results is that the majority of the detected motifs are target sites for regulatory factors involved in the control of cell cycle, development and differentiation. These findings support the existence of a complex interdependence among mitochondrial biogenesis and these biological processes as already suggested by different experimental works. A study carried out by Allombert-Blaise et al. [25] on the terminal differentiation of human epidermal keratinocytes reveals that one key step involves mitochondria-dependent cell death machinery through a spatial-temporal coordination of the differentiation program with the apoptotic machinery. Another recent study aiming to elucidate how the regulation of cell cycle is intimately linked to erythroid differentiation, has provided experimental evidences that retinoblastoma protein (Rb), a central regulator of the cell cycle, intrinsically promotes erythropoiesis by coupling cell cycle exit with mitochondrial biogenesis [26]. These findings demonstrate a strict correlation among mitochondrial biogenesis, cell cycle regulation and tissue differentiation and, in particular, they underline that these two different pathways, erythropoiesis and mitochondrial biogenesis, play a role in co-ordinately regulating cellular differentiation.

**Table 2:** Statistics of types of regulatory RNA motifs in UTRminer.

***Location (5' UTR)***	
***Regulatory motifs***	***Occurrences***
Upstream Open Reading Frame (uORF)	437
Internal Ribosome Entry Site (IRES)	324
Terminal Oligopyrimidine Tract (TOP)	48
UNR binding site (UNR-bs)	17
Iron Responsive Element (IRE)	8
S12 mitochondrial protein 5'UTR translation control element (RPMS12_TCE)	3
SXL binding site (SLX-bs)	2
Mos polyadenylation response element (Mos-PRE)	2
***Location (3' UTR)***	

Upstream Open Reading Frame (uORF)	1693
Polyadenylation Signal (PAS)	1361
Internal Ribosome Entry Site (IRES)	444
K-Box (KB)	177
SXL binding site (SLX-bs)	131
Brd-Box (Brd)	128
UNR binding sites (UNR-bs)	123
GY-Box (GY)	114
Mos polyadenylation response element (Mos-PRE)	54
Cytoplasmic polyadenylation element (CPE)	46
Alcohol dehydrogenase 3'UTR downregulation control element (ADH_DRE)	32
Selenocysteine Insertion Sequence – type 1 (SECIS1)	13
AU-rich class-2 Element (ARE2)	3
Insulin 3'UTR stability element (INS_SCE)	3
Bruno 3'UTR responsive element (BRE)	2
15-Lipoxygenase Differentiation Control Element (15-LOX-DICE)	2
Glusose transporter type-1 3'UTR cis-acting element (GLUT1)	1
Iron Responsive Element (IRE)	1

The table reports the standard name of regulatory RNA motifs and the total number of motifs' occurrences in the 5'- and 3' UTR collections.

Nature and distribution of regulatory motifs in UTR sequences of mitochondrial targeted mRNA, as discovered by our analysis, may represent a precious resource for the elucidation of co-regulated networks controlling mitochondrial biogenesis and its role in development, differentiation and cell cycle regulation. Some interesting examples are reported next.

### The two stepped data mining approach

The first step of the data mining analysis returns the frequent pattern (INIT) of associated motifs inside 5'- and 3'-UTR sequence collections as a combination of motifs at a different levels of complexity, then referred as "Pattern level" (PL). The basic level is level 1 (k = 1), i.e. how many frequent UTR motifs have been detected as a single occurrence. For each INIT, the system provides the χ^2 ^and α values returned by the statistical analysis. In this way, what is observed is how many times the motifs A is frequently found in association with the motif B (PL = 2) and how significant this result is, and then, how many times the same motifs (A+B) are still frequently found in association with a motif C (PL = 3) and so on.

This mining step returned several sets of frequent multiple occurrences of different motifs, along with their corresponding sets of supporting UTR sequences. Namely, we have obtained a total of 5 INITs in the 5' UTR sequence collection, and a total of 211 INITs in the 3' UTR collection. A summary of INITs produced by the analysis of the overall collection (M_dataset) is provided as additional file 1. The number and type of the different detected INITs reflect the different complexity of the two regions underlined in the previous paragraph.

Table 3 gives a detailed view on what features of FP are extracted by the system in this first step. UTRminer provides three different views: a compact, normal and full view. In this table, only one of the detected FPs is represented. It belongs to the class of the patterns at level 3 and is given by the co-occurrence of {PAS, IRES, uORF} motifs. This pattern is identified as INIT 127 in the M_dataset and as INIT 88 in the H_dataset. It has been detected in 320 sequences of the M_dataset and in 111 sequences of the H_dataset. The compact view shows that among all the supporting 3' UTR sequences, 296 (> 92%) in the M_dataset and 107 (> 96%) in the H_Dataset present the same spatial order of motifs, namely some occurrences of uORF are followed by some occurrences of IRES which are in turn followed by some occurrences of PAS. The exact number of occurrences of each motif in UTR sequences which support this pattern is provided by the "normal view" section. For instance, 47 occurrences of the pattern {uORF – uORF – IRES-PAS} have been found in the M_dataset. The sequence is compactly represented as follows: {uORF [2] IRES PAS}. A more detailed drill-down view, called "Full view", provides, for each mined pattern, the length of each motif, the starting and ending position of the motif inside the UTR sequence, the length of the spacer which separates a motif from the following one and the UTRef ID reference. The negative value of the spacer indicates to what extent a motif overlaps the closest one.

**Table 3:** An example of UTRminer FP views.

***mt-3' UTR Total Patterns***	***mt-3' UTR Human Patterns***
***Pattern level: 3***	

INIT: 127 | IRES, PAS, uORF Support items: 320 Chi-square: 9.88 | Significant (alpha = 0.05)	INIT: 88 | IRES, PAS, uORF- Support items: 111 Chi-square: 6.622 | Significant (alpha = 0.05)

***Positional patterns: compact view***	

* IRES uORF PAS (6)	* IRES uORF PAS (1)
* uORF IRES PAS (296)	* uORF IRES PAS (107)
* uORF IRES uORF PAS (18)	* uORF IRES uORF PAS (3)

***Positional pattern: normal view***	

*IRES uORF PAS (6)	* IRES uORF PAS (1)
* uORF IRES PAS (93)	* uORF IRES PAS (24)
* uORF IRES uORF PAS (6)	* uORF IRES uORF PAS (1)
* uORF[2] IRES PAS (47)	* uORF[2] IRES PAS (12)
* uORF[2] IRES uORF PAS (3)	* uORF[3] IRES PAS (10)
* uORF[3] IRES PAS (34)	* uORF[4] IRES PAS (8)
* uORF[3] IRES uORF PAS (3)	* uORF[5] IRES PAS (5)
*.......................	*......................

***Positional pattern: full view***	

* uORF (207:33..239) 11 | IRES (96:249..344) -20 | PAS (22:323..344) [CR655030]	* uORF (132:9..140) -9 | IRES (96:130..225) -33 | PAS (35:191..225) [CR042183]
*.............................................	*................................

The table shows the three views of UTRminer on results of the first step. Data refer to the specific example {PAS, IRES, uORF} in the M_dataset (mt-3' UTR Total Patterns) and in the H_dataset (mt-3' UTR Human Patterns). In the normal and full view, only few rows of the overall results are shown. Support items = number of UTR sequences bringing the RNA target sites.

In the second step each set of sequences featured by a single INIT have been analysed with GSP for the discovery of FSP. Experiments have been conducted by varying both the number of *bins *(6, 9, 12) for spacer discretization and the input threshold (*minsup *= 0.20 and 0.30) for minimum support of frequent patterns. Henceforth, we keep focusing on the FP {PAS, IRES, uORF} found at the first step because it is one of those with the greatest support and we report the results obtained at the second step for the two sets of supporting sequences (INIT 127 and INIT88). Statistics on mined FSPs are reported in Tables 4 and 5.

**Table 4:** FSPs discovered by running GSP on INIT 88

***Minsup***	***Pattern length***	***FSPs***		
		
		*6 bins*	*9 bins*	*12 bins*
0.2	1	9	12	15
	2	15	22	27
	3	17	11	15
	4	16	2	2
	5	5	1	1

0.3	1	7	11	14
	2	14	20	23
	3	8	11	12
	4	2	2	2
	5	1	1	1

Pattern length = number of pattern's elements, it includes regulatory RNA motifs and spacers.

**Table 5:** FSPs discovered by running GSP on INIT 127

***Minsup***	***Pattern length***	***FSPs***		
		
		*6 bins*	*9 bins*	*12 bins*
0.2	1	9	12	15
	2	15	19	20
	3	8	9	9
	4	2	2	2
	5	1	1	1

0.3	1	6	9	11
	2	15	16	17
	3	7	7	6
	4	2	2	2
	5	-	-	-

Pattern length = number of pattern's elements, including regulatory RNA motifs and spacers.

As expected, the number of FSPs detected decreases as the pattern length increases since long patterns are less common than short ones, although the real existence of more complex patterns may not be excluded. Factors influencing prediction of longer sequential patterns may be the size of the sample or an intrinsic inefficiency of the algorithm. Problems related to the size of the sample may be solved by lowering the input threshold for the minimum pattern support (*minsup*). In the case of our experimentation, we cannot exclude that a more comprehensive dataset than the one we used would have given better results. On the other hand, this prediction may also be affected by the fixed-width intervals determined by spacer discretization as well as by a computational inefficiency of GSP to handle very long sequential patterns.

The length of discovered patterns is negatively correlated with the granularity of the discretization. Indeed, by increasing the number of *bins*, FSPs are less supported and it is more likely that a long pattern (in terms of motifs and spacers) is missed. Table 6 compares FSPs discovered at PL5 for different discretizations (6, 9 and 12 *bins*) and for two different *minsup *values (0.20 and 0.30). Among all possible combinations, the most frequent are represented by patterns where the 3' region of uORF partially overlaps the 5' region of the IRES motif (Table 6). The comparison of results at different *bins *gives a different outcome in respect of spacers length and numbers of supporting items, demonstrating that increasing the granularity of the discretization it is possible to improve the specificity of the instrumental analysis. At *bins *= 6, the most frequent pattern is <uORF {-99-(-18.5)} IRES {-99-(-18.5)} PAS > and it occurs in 47 out of 111 UTRs (42.34%) in the H_dataset (INIT 88) and in 89 out of 320 UTRs (27,8%) in the M_dataset (INIT 127). These results demonstrate that the evaluation of spacer conservation improve the quality of prediction compared to the first step of the mining process, where no evaluation about structural features of spacers is done. A further stringency is introduced by increasing the sensibility of the analysis through the refinement of the spacer discretization. Indeed, when the analysis is carried out at granularity of 9 and 12 *bins*, the size of the support further decreases, accounting for the 30,6% of the human sequences and for the 10,6% of the total one. The difference between the prediction at 9 and at 12 *bins *consists in a reduction of the spacers width, while no difference can be detected between the prediction at *minsup *0.2 and 0.3.

**Table 6:** Results of the second mining step on INIT 88 and INIT 127.

***INIT 88***		
	***minsup 0.2***	***minsup 0.3***

***bin 6***	uORF {-99-(-18.5)} IRES {-99-(-18.5)} PAS > (support:47)	uORF {-99-(-18.5)} IRES {-99-(-18.5)} PAS > (support:47)
***bin 9***	uORF {-99-(-25.5)} IRES {-25.5-0.5}PAS > (support: 34)	uORF {-99-(-25.5)} IRES {-25.5-0.5} PAS > (support:34)
***bin 12***	uORF {-99-(-30.5)} IRES {-30.5-(-18.5)} PAS > (support:34)	uORF {-99-(-30.5)} IRES {-30.5-(-18.5)} PAS> (support:34)

***INIT 127***		

	***minsup 0.2***	***minsup 0.3***

***bin 6***	uORF {-99-(-22.5)} IRES {-22.5-4.5} PAS > (support:89)	-
***bin 9***	uORF{-99-(-30.5)} IRES {-30.5-(-17.5)} PAS > (support:89)	-
***bin 12***	uORF {-99-36.5} IRES {-22.5-(-14.5)} PAS > (support:64)	-

Support = number of UTR sequences matching the constraints expressed by the FSP.

By enlarging the dataset from the human to the total sample the prediction is confirmed at *minsup *= 0.2 but not at *minsup *= 0.3. This difference can be explained by the heterogeneity of species represented in M_dataset, as well as by the skewed distribution of the species (human annotated sequences are 37% of the M_dataset). Another important issue to be considered is that more different the species are, the less likely they share the same spatial pattern for a set of motifs. A stronger representation of species belonging to the same taxa could compensate this bias allowing to compare gene-specific patterns of regulatory motifs to a larger extension. Poor annotations availability for species studied at a lesser extent than the human one could also contribute to this bias since no orthologs in the other species could be found in many cases. To correct this bias it is necessary to augment the dataset with new and more complete annotations. The more species-specific the dataset is, the more effective predictions can be obtained. This does not mean that a more general application of the method to heterogeneous datasets cannot be envisaged, but that in these cases lower *minsup *thresholds should be considered in order to prevent the loss of patterns which look infrequent for underrepresented species.

### Some interesting insights on regulatory RNA target sites co-occurrences detected in the 3'-UTRs of mitochondrial transcripts

Table 7 reports a synthesis of distribution of UNR-bs and SLX-bs homologs in the 3' UTR M_dataset. Here we also report their association with other regulatory target sites detected along the same UTR sequences.

**Table 7:** UNR-bs and SLX-bs FPs in UTRminer 3' UTRs.

***INIT***	***PL***	***Frequent Pattern***							***%***
24	2	UNR-bs	SXL-bs						0.82%
87	3	UNR-bs	SXL-bs				uORF		0.82%
79	3	UNR-bs	SXL-bs					PAS	0.56%
156	4	UNR-bs	SXL-bs				uORF	PAS	0.56%
195	4	UNR-bs	SXL-bs	KB			uORF		0.26%
92	3	UNR-bs	SXL-bs			Mos-PRE			0.26%
165	4	UNR-bs	SXL-bs			Mos-PRE	uORF		0.26%
161	4	UNR-bs	SXL-bs	KB				PAS	0.2%
211	5	UNR-bs	SXL-bs	KB			uORF	PAS	0.2%
53	3	UNR-bs	SXL-bs		Brd				0.2%
138	4	UNR-bs	SXL-bs		Brd		uORF		0.2%

The order in which RNA target sites are listed in table rows is neither indicative of the order of the target sites along the UTR sequences, nor indicative of the multiple target site occurrence. This information can be retrieved in the "Frequent Patterns" Section of UTRminer. PL = Pattern level, % = percentage of sequences supporting the FSP.

SLX-bs is a conserved U-rich target site for the binding of the Sex-lethal (SXL) protein in both the 5'- and 3'-UTRs of male-specific-lethal 2 mRNA (msl-2) in Drosophila [27]. The binding of SLX to msl-2 is essential to repress the assembly of the dosage compensation complex (DCC) and the subsequent expression of X-linked genes as a part of the dosage compensation process [28]. A factor necessary for SLX-mediated repression is the protein upstream of N-ras (UNR), an ubiquitous protein that is recruited to the 3' UTR of msl-2 by SXL [29]. UNR is a cytoplasmic RNA-binding protein, widely investigated in mammals, involved in the regulation of messenger RNA stability and internal initiation of translation [30-32]. UNR has a key role in cell proliferation and response to stress, two cell processes, which are strictly related to mitochondrial functions. Analysing the distribution of UNR-bs and SXL-bs homologs in our sample we found that both these motifs occur in multiple copies along the same UTR sequences and are frequently associated. They are located on different types of transcripts in *H. sapiens*, *M. musculus*, *R. norvegicus*, *B. taurus *and *D. rerio*. Some are on the X chromosome, such as the *ca5b *and *slca6 *genes in *H. sapiens *and the *Maoa *and *Acsl4 *(isoform 1 and 2) genes in *M. musculus *(information extracted by the analysis of "INIT 24" and "125" in the "mt-3'UTR total pattern section of UTRminer). The function of these genes is different from the one of *msl-2 *in Drosophila, nevertheless, this findings could open new interesting perspectives of investigation. The first one is that, in mammals, the dosage compensation of some X-linked genes could be mediated at a translational level by the direct inhibition of transcripts with a mechanism similar to the one adopted in Drosophila for *mls-2*. Secondly, the concerted action of SLX and UNR may not be limited to their role in the X-chromosome dosage compensation process but also used to coordinate nuclear and mitochondrial genome expression in specific steps of development and cell proliferation as well as under stressing conditions. Moreover, we find SLX and UNR target sites in multiple association with other regulatory motifs involved in embryo development and differentiation such as the miRNA target sites (Brd-box, GY-box and K-box) and Mos polyadenylation response element (Mos-PRE). These findings support the hypothesis suggested by Abaza and F. Gebauer [33] about additional interactions of SLX and UNR with other translation repressors. Furthermore, their wide distribution in transcript for mitochondrial proteins also suggests a potential role of these translation regulatory factors in mitochondrial biogenesis and function.

In many of the FPs discovered, Brd-, GY- and K-box are often present in multiple copies and frequently associated in different combination along the same transcripts. Unfortunately, due to the limited size of our sample, the small number of sequences associated to each of the discovered FP does not allow us to have a support sufficiently large to extract significant information about conservation of spacing among motif occurrences. Nevertheless, these findings support the possibility that predicted motifs could actually operate in synergy to fine-tune transcript expression during differentiation and development as suggested by experimental studies in Drosophila, which support the idea of a synergic effect between miRNA-binding sites on the same transcript [34]. In addition and more in general, these findings provide new interesting clues for the elucidation of mechanisms used by both miRNA and SLX-UNR to mediate translation inhibition.

### UTRminer web site

The UTRminer web interface allows users to access data on FPs and FSPs detected through a menu on the left of the home page. This menu provides the access to three different sections, "Frequent Patterns", "Frequent Sequential Patterns" and "Databases".

The "Frequent Patterns" section collects and shows the results of the first mining step, which is a list of INITs representing FPs of motifs at increasing levels of complexity. For each one of the listed INIT, the system provides the access to a compact, normal and full view, as already described in the previous paper section, and allows the retrieval of UTR sequences supporting the FP.

The "Frequent Sequential Patterns" section shows the results of the second mining step on INITs collected in the "Frequent Patterns" section. Here, for each INIT, UTRminer provides the list of "Generalized Sequential Patterns" at a different *minsup *and *bin*, reported in square brackets on the right of each pattern. As an example, in the following numerical sequence " [3 6 0.2]" the first item is the INIT number which identifies the FP, the second item is the value of *bins *and the third one is the *minsup *threshold used for the analysis. Results of the second mining step can also be accessed from the "Frequent Patterns" pages by clicking on the INIT units.

In each section, UTRminer separately collects mined patterns in two groups, "5'UTR" and "3'UTR" each one still grouped in two different sections, one collecting results from the analysis of all species (Total patterns), and the other one collecting results on the human subset (Human patterns). For each pattern, the list of supporting sequences (UTRef ID) and the direct link to the UTRminer entries are provided. This list can be used in the UTRminer "Databases" section to browse information about sequences supporting patterns in the different related databases.

The "Databases" section allows the user to browse data collected and integrated from the UTRef, UTRsite and MitoRes databases as well as data annotated in UTRminer by using one or a list of sequence IDs. This search option gives back a predefined list of information in two different formats, "List" and "Table". The pieces of information provided by browsing the UTRminer database are the annotated motifs and their position on the UTR sequences along with their distances from the CDS and hyperlink to the UTRef and MitoRes database entries. Searching the UTRef and MitoRes databases, the returned information are species, sequence function, UTR length, gene name and protein along with the sequence ID and hyperlinks to related databases such RefSeq, PFam and GO. The UTRsite database can be browsed by using the ID or the name of the motif and information provided are the standard name of the motif and its description along with the hyperlink to the UTRsite entry.

## Conclusion

The discovery of frequent co-occurrences of mRNA regulatory motifs may reveal new insights about their mechanism of action and their likely roles in post-transcriptional control of gene expression. The development of UTRminer significantly contributes to this aim and opens the way to new challenging perspectives. The reported experimentation of UTRminer was carried out on a collection of UTR sequences from nuclear mRNAs targeting the mitochondria. The survey on nature and distribution of UTR regulatory motifs detected in our dataset has allowed us to highlight some interesting structural features which suggest complex and multiple interactions of regulatory factors controlling translation. These factors are involved in the control of key steps of cell cycle, development and differentiation, and, as highlighted by recent studies, they prove the existence of a complex interdependence between mitochondrial biogenesis and these biological processes. These findings may represent reliable clues for experimental validations that could contribute to the elucidation of both mechanisms underlying regulated translation and its role in controlling mitochondrial biogenesis.

From a computational point of view, the contribution of this work is mainly based on the use of sequential pattern miners, which allow to overcome some limits of other competitive tools. The discovery process is exclusively based on structural properties of sequences, with no assumption on the number and the nature of motifs as well as on their spatial displacement. Moreover, most of the motif finders developed up to date work under the assumption that the length of a spacer between two consecutive motifs remains rigidly fixed for the same couple of motifs. This means that two motifs of the same pattern, even though differently spaced by few nucleotides, are considered as two different spaced patterns. UTRminer overcomes this limit by introducing a certain tolerance in the width of the spacer between two motifs along the same pattern, since the length of spacers is discretized before mining FSP.

The number of FSPs discovered by UTRminer depends on two parameters: the input threshold of the minimum pattern support and the granularity of the discretization of spacers length. By varying these two parameters it is possible to flexibly customize the tool in order to discover conserved FSPs of motifs both in species-specific and in heterogeneous datasets. Obviously the greater the species coverage of analyzed sequences, the better the result is.

FPs identified by the reported UTRminer analysis amount to 216 and can be retrieved at the UTRminer web site. Here a series of search options allows the user to browse the resource at different levels. As an overall conclusion we can say that the results obtained in this preliminary study are encouraging for both the reliability of the computational approach used and for the structural features of UTR regulatory motifs distribution it has allowed to highlight.

Future work will include: i) the updating of the UTR sequence collections on the most recent RefSeq release; ii) the enlargement of the UTR sequence collections to as many as possible species; iii) the enlargement of the regulatory motif collection with miRNA target site collections from specialized databases; iv) the improvement of the second mining step to increase the capability of the process to discover longer sequential patterns, by applying an alternative method based on our previous work on spatial association rule mining [35]; v) the addition of positional and functional correlation of frequent predicted sequential patterns with data on expression of target genes.

## Competing interests

The authors declare that they have no competing interests.

## Authors' contributions

DD proposed and coordinated the work, and did the biological analysis. AT defined the first data mining step, implemented the respective Java application and produced the corresponding results. GG designed and implemented the UTRminer database, the pipelines for the extraction of data from external resources, interfaced the UTRminer database via web and made the experimental results available in the UTRminer website. ES, CL and DM defined the second mining step and produced the corresponding experimental results. CL, ES, DM and DD wrote the manuscript.

## Supplementary Material

Additional file 1 **Results of the first mining step on regulatory RNA motifs in UTRminer**. Data reported in the table gives a general overview of results of the first mining step runs. The percentage of each INIT detected in respect of the total sample is shown. The order of RNA target sites in table INIT rows, is neither indicative of the order of the target sites along the UTR sequences, nor indicative about the presence of multiple copies of the same target site. PL = Pattern level, % = percentage of sequences supporting the FSP. Click here for file
