# Supplementary material for: Computational annotation of UTR cis-regulatory modules through Frequent Pattern Mining
Source: BMC Bioinformatics. 2009 Jun 16;10(Suppl 6):S25. doi: 10.1186/1471-2105-10-S6-S25 (PMC2697649; doi:10.1186/1471-2105-10-S6-S25)
Supplement: Additional file 1 — Results of the first mining step on regulatory RNA motifs in UTRminer. Data reported in the table gives a general overview of results of the first mining step runs. The percentage of each INIT detected in respect of the total sample is shown. The order of RNA target sites in table INIT rows, is neither indicative of the order of the target sites along the UTR sequences, nor indicative about the presence of multiple copies of the same target site. PL = Pattern level, % = percentage of sequences supporting the FSP. [file 1471-2105-10-S6-S25-S1.doc]

**Addtional file 1 – Results of the first mining step on regulatory RNA motifs in UTRminer**

Data reported in the table gives a general overview of results of the first mining step runs. The percentage of each INIT detected in respect of the total sample is shown. The order of RNA target sites in table INIT rows, is neither indicative of the order of the target sites along the UTR sequences, nor indicative about the presence of multiple copies of the same target site. PL = Pattern level, % = percentage of sequences supporting the FSP.

| *INIT* | *PL* | ***Frequent pattern in 5’-UTRs*** | | | | | | | | | | | | *%* |
| --- | --- | --- | --- | --- | --- | --- | --- | --- | --- | --- | --- | --- | --- | --- |
| 3 | 2 |  |  |  |  |  |  | UNR-bs |  |  |  | uORF |  | 0.72% |
| 4 | 2 | TOP |  |  |  |  |  |  |  |  | IRES |  |  | 0.57% |
| 5 | 2 | TOP |  |  |  |  |  |  |  |  |  | uORF |  | 0.57% |
| 6 | 2 |  |  |  |  |  |  |  |  |  | IRES | uORF |  | 5.76% |
| 8 | 3 | TOP |  |  |  |  |  |  |  |  | IRES | uORF |  | 0.26% |
|  |  | ***Frequent pattern in 3’-UTRs*** | | | | | | | | | | | |  |
| 1 | 2 | ADH_DRE | GY |  |  |  |  |  |  |  |  |  |  | 0.2% |
| 2 | 2 |  | GY | SECIS1 |  |  |  |  |  |  |  |  |  | 0.2% |
| 3 | 2 | ADH_DRE |  |  | KB |  |  |  |  |  |  |  |  | 0.2% |
| 4 | 2 | ADH_DRE |  |  |  | SXL-bs |  |  |  |  |  |  |  | 0.41% |
| 5 | 2 |  | GY |  |  |  | Mos-PRE |  |  |  |  |  |  | 0.26% |
| 6 | 2 | ADH_DRE |  |  |  |  |  | UNR-bs |  |  |  |  |  | 0.36% |
| 7 | 2 |  |  |  |  |  |  |  | Brd | CPE |  |  |  | 0.26% |
| 8 | 2 |  |  |  |  | SXL-bs |  |  | Brd |  |  |  |  | 0.92% |
| 9 | 2 |  |  |  |  |  | Mos-PRE |  | Brd |  |  |  |  | 0.46% |
| 10 | 2 |  |  |  |  | SXL-bs |  |  |  | CPE |  |  |  | 0.41% |
| 11 | 2 |  |  |  |  |  |  |  |  | CPE | IRES |  |  | 0.31% |
| 12 | 2 |  |  | SECIS1 |  |  |  |  |  |  |  |  | PAS | 0.36% |
| 13 | 2 |  | GY |  |  |  |  |  | Brd |  |  |  |  | 0.77% |
| 14 | 2 |  |  |  |  |  |  | UNR-bs | Brd |  |  |  |  | 0.97% |
| 15 | 2 | ADH_DRE |  |  |  |  |  |  |  |  | IRES |  |  | 0.26% |
| 16 | 2 |  |  |  |  |  |  |  | Brd |  |  |  | PAS | 4.82% |
| 17 | 2 |  |  |  |  |  |  |  |  | CPE |  |  | PAS | 2.05% |
| 18 | 2 |  |  |  |  |  | Mos-PRE |  |  |  | IRES |  |  | 1.02% |
| 19 | 2 |  | GY |  |  |  |  |  |  |  | IRES |  |  | 1.08% |
| 20 | 2 |  |  |  |  |  | Mos-PRE | UNR-bs |  |  |  |  |  | 0.97% |
| 21 | 2 |  |  |  | KB |  | Mos-PRE |  |  |  |  |  |  | 0.67% |
| 22 | 2 |  |  |  |  | SXL-bs | Mos-PRE |  |  |  |  |  |  | 0.77% |
| 23 | 2 |  | GY |  |  |  |  | UNR-bs |  |  |  |  |  | 0.67% |
| 24 | 2 |  |  |  |  | SXL-bs |  | UNR-bs |  |  |  |  |  | 0.82% |
| 25 | 2 |  | GY |  |  | SXL-bs |  |  |  |  |  |  |  | 1.02% |
| 26 | 2 |  | GY |  |  |  |  |  |  |  |  |  | PAS | 4.1% |
| 27 | 2 |  |  |  | KB |  |  |  |  |  |  |  | PAS | 6.3% |
| 28 | 2 |  |  |  |  | SXL-bs |  |  |  |  |  |  | PAS | 4.82% |
| 29 | 2 |  |  |  |  |  |  |  |  |  | IRES | uORF |  | 19.52% |
| 30 | 2 |  |  |  |  |  | Mos-PRE |  |  |  |  | uORF |  | 2.72% |
| 31 | 2 |  |  | SECIS1 |  |  |  |  |  |  |  | uORF |  | 0.67% |
| 32 | 2 |  |  |  |  |  |  | UNR-bs |  |  |  | uORF |  | 6.2% |
| 33 | 2 |  | GY |  |  |  |  |  |  |  |  | uORF |  | 5.79% |
| 34 | 2 |  |  |  | KB |  |  |  |  |  |  | uORF |  | 8.76% |
| 35 | 2 |  |  |  |  | SXL-bs |  |  |  |  |  | uORF |  | 6.61% |
| 36 | 2 |  |  |  |  |  |  |  |  | CPE |  | uORF |  | 2.2% |
| 37 | 2 |  |  |  |  |  |  | UNR-bs |  |  | IRES |  |  | 1.18% |
| 38 | 2 |  |  |  | KB |  |  |  | Brd |  |  |  |  | 1.54% |
| 39 | 2 |  |  |  | KB |  |  |  |  |  | IRES |  |  | 2.1% |
| 40 | 2 |  |  |  |  |  |  |  | Brd |  | IRES |  |  | 2.15% |
| 41 | 2 |  |  |  |  | SXL-bs |  |  |  |  | IRES |  |  | 1.79% |
| 42 | 2 |  |  |  |  |  |  |  |  |  |  | uORF | PAS | 61.01% |
| 43 | 2 | ADH_DRE |  |  |  |  |  |  |  |  |  |  | PAS | 0.92% |
| 44 | 2 |  |  |  |  |  |  |  |  |  | IRES |  | PAS | 18.8% |
| 45 | 2 | ADH_DRE |  |  |  |  |  |  |  |  |  | uORF |  | 1.64% |
| 46 | 2 |  |  |  |  |  | Mos-PRE |  |  |  |  |  | PAS | 2.05% |
| 47 | 2 |  |  |  | KB |  |  | UNR-bs |  |  |  |  |  | 1.23% |
| 48 | 2 |  |  |  |  |  |  |  | Brd |  |  |  |  | 6.51% |
| 49 | 2 |  | GY |  | KB |  |  |  |  |  |  | uORF |  | 1.33% |
| 50 | 2 |  |  |  |  |  |  | UNR-bs |  |  |  |  | PAS | 4.87% |
| 51 | 2 |  |  |  | KB | SXL-bs |  |  |  |  |  |  |  | 1.38% |
| 52 | 3 |  |  |  |  |  |  |  | Brd | CPE |  |  | PAS | 0.2% |
| 53 | 3 |  |  |  |  | SXL-bs |  | UNR-bs | Brd |  |  |  |  | 0.2% |
| 54 | 3 | ADH_DRE |  |  |  |  |  | UNR-bs |  |  |  |  | PAS | 0.2% |
| 55 | 3 |  | GY | SECIS1 |  |  |  |  |  |  |  | uORF |  | 0.2% |
| 56 | 3 |  | GY |  |  | SXL-bs |  |  | Brd |  |  |  |  | 0.2% |
| 57 | 3 |  | GY |  |  |  | Mos-PRE |  |  |  |  |  | PAS | 0.2% |
| 58 | 3 | ADH_DRE | GY |  |  |  |  |  |  |  |  | uORF |  | 0.2% |
| 59 | 3 |  |  |  |  |  | Mos-PRE |  | Brd |  | IRES |  |  | 0.26% |
| 60 | 3 |  | GY |  |  | SXL-bs |  |  |  |  | IRES |  |  | 0.2% |
| 61 | 3 |  | GY |  | KB |  |  | UNR-bs |  |  |  |  |  | 0.2% |
| 62 | 3 |  |  |  |  |  |  |  | Brd | CPE |  | uORF |  | 0.26% |
| 63 | 3 |  |  |  | KB | SXL-bs |  |  | Brd |  |  |  |  | 0.36% |
| 64 | 3 | ADH_DRE |  |  |  | SXL-bs |  |  |  |  |  |  | PAS | 0.36% |
| 65 | 3 | ADH_DRE |  |  |  |  |  | UNR-bs |  |  |  | uORF |  | 0.36% |
| 66 | 3 | ADH_DRE |  |  | KB |  |  |  |  |  |  | uORF |  | 0.2% |
| 67 | 3 |  |  |  |  |  |  |  |  | CPE | IRES |  | PAS | 0.31% |
| 68 | 3 |  | GY |  |  |  | Mos-PRE |  |  |  |  | uORF |  | 0.26% |
| 69 | 3 |  |  |  |  |  |  |  |  | CPE | IRES | uORF |  | 0.31% |
| 70 | 3 |  | GY |  |  |  |  | UNR-bs | Brd |  |  |  |  | 0.31% |
| 71 | 3 |  |  |  | KB |  |  | UNR-bs | Brd |  |  |  |  | 0.26% |
| 72 | 3 |  | GY |  | KB | SXL-bs |  |  |  |  |  |  |  | 0.2% |
| 73 | 3 |  |  |  |  |  | Mos-PRE |  | Brd |  |  | uORF |  | 0.46% |
| 74 | 3 |  | GY |  | KB |  |  |  | Brd |  |  |  |  | 0.31% |
| 75 | 3 |  |  |  |  | SXL-bs |  |  |  |  | IRES |  | PAS | 1.69% |
| 76 | 3 |  | GY |  |  |  |  |  | Brd |  |  | uORF |  | 0.77% |
| 77 | 3 |  |  |  |  | SXL-bs | Mos-PRE |  |  |  |  |  | PAS | 0.46% |
| 78 | 3 |  | GY |  |  |  |  | UNR-bs |  |  |  |  | PAS | 0.46% |
| 79 | 3 |  |  |  |  | SXL-bs |  | UNR-bs |  |  |  |  | PAS | 0.56% |
| 80 | 3 |  | GY |  |  | SXL-bs |  |  |  |  |  |  | PAS | 0.56% |
| 81 | 3 |  |  |  |  | SXL-bs |  |  | Brd |  |  | uORF |  | 0.92% |
| 82 | 3 | ADH_DRE |  |  |  |  |  |  |  |  | IRES | uORF |  | 0.26% |
| 83 | 3 |  |  |  |  |  | Mos-PRE | UNR-bs |  |  |  | uORF |  | 0.97% |
| 84 | 3 |  |  |  | KB |  |  |  | Brd |  | IRES |  |  | 0.51% |
| 85 | 3 | ADH_DRE |  |  |  | SXL-bs |  |  |  |  |  | uORF |  | 0.41% |
| 86 | 3 |  |  |  |  | SXL-bs |  |  |  | CPE |  |  | PAS | 0.41% |
| 87 | 3 |  |  |  |  | SXL-bs |  | UNR-bs |  |  |  | uORF |  | 0.82% |
| 88 | 3 |  |  |  |  | SXL-bs |  |  |  | CPE |  | uORF |  | 0.36% |
| 89 | 3 |  |  |  |  | SXL-bs |  |  | Brd |  | IRES |  |  | 0.46% |
| 90 | 3 |  | GY |  | KB |  |  |  |  |  | IRES |  |  | 0.41% |
| 91 | 3 |  |  |  | KB |  | Mos-PRE | UNR-bs |  |  |  |  |  | 0.31% |
| 92 | 3 |  |  |  |  | SXL-bs | Mos-PRE | UNR-bs |  |  |  |  |  | 0.26% |
| 94 | 3 |  |  |  | KB |  |  |  |  |  |  | uORF | PAS | 6.1% |
| 95 | 3 |  |  |  |  | SXL-bs |  |  |  |  |  | uORF | PAS | 4.71% |
| 96 | 3 |  |  |  |  |  | Mos-PRE |  |  |  | IRES |  | PAS | 0.92% |
| 97 | 3 |  |  |  |  |  |  | UNR-bs |  |  | IRES |  | PAS | 1.08% |
| 98 | 3 |  | GY |  |  |  |  |  |  |  | IRES |  | PAS | 0.97% |
| 99 | 3 |  |  |  |  |  |  |  | Brd |  |  | uORF | PAS | 4.76% |
| 100 | 3 |  |  |  | KB |  |  |  |  |  | IRES |  | PAS | 1.84% |
| 101 | 3 | ADH_DRE |  |  |  |  |  |  |  |  |  | uORF | PAS | 0.92% |
| 102 | 3 |  |  |  |  |  | Mos-PRE | UNR-bs |  |  |  |  | PAS | 0.77% |
| 103 | 3 |  |  |  |  |  |  | UNR-bs | Brd |  |  | uORF |  | 0.97% |
| 104 | 3 |  |  |  | KB |  | Mos-PRE |  |  |  |  |  | PAS | 0.56% |
| 105 | 3 |  |  |  |  |  |  | UNR-bs | Brd |  |  |  | PAS | 0.72% |
| 106 | 3 |  |  |  | KB |  |  | UNR-bs |  |  |  |  | PAS | 0.97% |
| 107 | 3 |  |  |  | KB |  |  |  | Brd |  |  | uORF |  | 1.54% |
| 108 | 3 |  | GY |  | KB |  |  |  |  |  |  |  | PAS | 0.87% |
| 109 | 3 |  | GY |  |  |  |  |  | Brd |  |  |  | PAS | 0.72% |
| 110 | 3 |  |  |  | KB | SXL-bs |  |  |  |  |  |  | PAS | 1.02% |
| 111 | 3 |  |  |  |  |  | Mos-PRE |  |  |  | IRES | uORF |  | 0.97% |
| 112 | 3 |  |  |  |  |  |  | UNR-bs |  |  | IRES | uORF |  | 1.18% |
| 113 | 3 |  | GY |  |  |  |  |  |  |  | IRES | uORF |  | 1.08% |
| 114 | 3 |  |  |  | KB |  |  |  |  |  | IRES | uORF |  | 2.1% |
| 115 | 3 |  |  |  |  | SXL-bs |  |  |  |  | IRES | uORF |  | 1.79% |
| 116 | 3 |  |  |  |  |  |  |  | Brd |  | IRES |  | PAS | 1.79% |
| 117 | 3 |  |  |  |  |  |  |  |  | CPE |  | uORF | PAS | 1.95% |
| 118 | 3 |  |  |  | KB |  | Mos-PRE |  |  |  |  | uORF |  | 0.67% |
| 119 | 3 |  |  |  |  | SXL-bs |  |  | Brd |  |  |  | PAS | 0.82% |
| 120 | 3 |  |  |  |  | SXL-bs | Mos-PRE |  |  |  |  | uORF |  | 0.77% |
| 121 | 3 |  | GY |  |  |  |  | UNR-bs |  |  |  | uORF |  | 0.67% |
| 122 | 3 |  |  |  | KB |  |  | UNR-bs |  |  |  | uORF |  | 1.23% |
| 123 | 3 |  |  |  | KB |  |  |  | Brd |  |  |  | PAS | 1.23% |
| 124 | 3 |  | GY |  | KB |  |  |  |  |  |  | uORF |  | 1.33% |
| 125 | 3 |  | GY |  |  | SXL-bs |  |  |  |  |  | uORF |  | 1.02% |
| 126 | 3 |  |  |  | KB | SXL-bs |  |  |  |  |  | uORF |  | 1.38% |
| 127 | 3 |  |  |  |  |  |  |  |  |  | IRES | uORF | PAS | 16.39% |
| 128 | 3 |  |  |  |  |  | Mos-PRE | UNR-bs |  |  | IRES |  |  | 0.51% |
| 129 | 3 |  |  |  |  |  | Mos-PRE |  |  |  |  | uORF | PAS | 2.05% |
| 130 | 3 |  |  |  | KB | SXL-bs |  |  |  |  | IRES |  |  | 0.41% |
| 131 | 3 |  |  |  |  |  |  |  | Brd |  | IRES | uORF |  | 2.15% |
| 132 | 3 |  |  | SECIS1 |  |  |  |  |  |  |  | uORF | PAS | 0.36% |
| 133 | 3 |  |  |  |  |  | Mos-PRE | UNR-bs |  |  |  | uORF | PAS | 4.82% |
| 134 | 3 |  |  |  | KB | SXL-bs |  |  |  |  |  |  |  | 0.26% |
| 135 | 3 |  | GY |  |  |  |  |  |  |  |  | uORF | PAS | 4.1% |
| 136 | 3 |  |  |  |  |  | Mos-PRE |  | Brd |  |  |  | PAS | 0.36% |
| 137 | 4 |  |  |  |  |  |  |  | Brd | CPE |  | uORF | PAS | 0.2% |
| 138 | 4 |  |  |  |  | SXL-bs |  | UNR-bs, | Brd |  |  | uORF |  | 0.2% |
| 139 | 4 |  | GY |  |  | SXL-bs |  |  | Brd |  |  |  | PAS | 0.2% |
| 140 | 4 |  | GY |  |  |  | Mos-PRE |  |  |  |  | uORF | PAS | 0.2% |
| 141 | 4 |  | GY |  |  | SXL-bs |  |  | Brd |  |  | uORF |  | 0.2% |
| 142 | 4 | ADH_DRE |  |  |  |  |  | UNR-bs |  |  |  | uORF | PAS | 0.2% |
| 143 | 4 |  | GY |  | KB |  |  | UNR-bs |  |  |  | uORF |  | 0.2% |
| 144 | 4 |  |  |  |  |  | Mos-PRE |  | Brd |  | IRES | uORF |  | 0.26% |
| 145 | 4 |  | GY |  |  |  |  | UNR-bs | Brd |  |  | uORF |  | 0.31% |
| 146 | 4 |  | GY |  |  |  |  | UNR-bs | Brd |  |  |  | PAS | 0.26% |
| 147 | 4 |  | GY |  |  | SXL-bs |  |  |  |  | IRES |  | PAS | 0.2% |
| 148 | 4 |  |  |  | KB |  | Mos-PRE | UNR-bs |  |  |  |  | PAS | 0.2% |
| 149 | 4 |  | GY |  |  | SXL-bs |  |  |  |  | IRES | uORF |  | 0.2% |
| 150 | 4 |  |  |  | KB |  |  | UNR-bs | Brd |  |  |  | PAS | 0.26% |
| 151 | 4 |  | GY |  | KB | SXL-bs |  |  |  |  |  | uORF |  | 0.2% |
| 152 | 4 |  |  |  |  | SXL-bs |  |  |  |  | IRES | uORF | PAS | 1.69% |
| 153 | 4 |  |  |  | KB |  |  |  | Brd |  | IRES |  | PAS | 0.51% |
| 154 | 4 |  |  |  |  | SXL-bs | Mos-PRE |  |  |  |  | uORF | PAS | 0.46% |
| 155 | 4 |  |  |  |  | SXL-bs |  |  | Brd |  | IRES | uORF |  | 0.46% |
| 156 | 4 |  |  |  |  | SXL-bs |  | UNR-bs |  |  |  | uORF | PAS | 0.56% |
| 157 | 4 |  |  |  | KB |  |  | UNR-bs | Brd |  |  | uORF |  | 0.26% |
| 158 | 4 |  | GY |  | KB |  |  |  |  |  | IRES |  | PAS | 0.31% |
| 159 | 4 |  |  |  |  |  | Mos-PRE |  | Brd |  | IRES |  | PAS | 0.26% |
| 160 | 4 |  |  |  | KB | SXL-bs | Mos-PRE |  |  |  |  |  | PAS | 0.2% |
| 161 | 4 |  |  |  | KB | SXL-bs |  | UNR-bs |  |  |  |  | PAS | 0.2% |
| 162 | 4 |  |  |  |  |  |  |  |  | CPE | IRES | uORF | PAS | 0.31% |
| 163 | 4 |  |  |  | KB |  | Mos-PRE | UNR-bs |  |  |  | uORF |  | 0.31% |
| 164 | 4 |  |  |  | KB | SXL-bs | Mos-PRE |  |  |  |  | uORF |  | 0.26% |
| 165 | 4 |  |  |  |  | SXL-bs | Mos-PRE | UNR-bs |  |  |  | uORF |  | 0.26% |
| 166 | 4 |  |  |  | KB | SXL-bs |  |  | Brd |  |  |  | PAS | 0.31% |
| 167 | 4 |  | GY |  |  |  |  |  |  |  | IRES | uORF | PAS | 0.97% |
| 168 | 4 |  |  |  | KB |  |  |  |  |  | IRES | uORF | PAS | 1.84% |
| 169 | 4 |  |  |  |  |  |  |  | Brd |  | IRES | uORF | PAS | 1.79% |
| 170 | 4 |  |  |  |  |  | Mos-PRE | UNR-bs |  |  |  | uORF | PAS | 0.77% |
| 171 | 4 |  |  |  | KB |  |  |  | Brd |  | IRES | uORF |  | 0.51% |
| 172 | 4 |  |  |  |  |  |  | UNR-bs | Brd |  |  | uORF | PAS | 0.72% |
| 173 | 4 |  |  |  |  |  | Mos-PRE |  |  |  |  | uORF | PAS | 0.56% |
| 174 | 4 |  | GY |  | KB |  |  | UNR-bs |  |  |  | uORF | PAS | 0.46% |
| 175 | 4 |  |  |  | KB |  |  | UNR-bs |  |  |  | uORF | PAS | 0.97% |
| 176 | 4 |  | GY |  |  |  |  |  | Brd |  |  | uORF | PAS | 0.72% |
| 177 | 4 |  | GY |  | KB |  |  |  |  |  |  | uORF | PAS | 0.87% |
| 178 | 4 |  | GY |  |  | SXL-bs |  |  |  |  |  | uORF | PAS | 0.56% |
| 179 | 4 |  |  |  | KB | SXL-bs |  |  |  |  |  | uORF | PAS | 1.02% |
| 180 | 4 |  |  |  |  |  | Mos-PRE | UNR-bs |  |  | IRES |  | PAS | 0.51% |
| 181 | 4 |  |  |  |  | SXL-bs |  |  | Brd |  | IRES |  | PAS | 0.46% |
| 182 | 4 |  | GY |  | KB |  |  |  | Brd |  |  | uORF |  | 0.31% |
| 183 | 4 |  |  |  | KB | SXL-bs |  |  |  |  | IRES |  | PAS | 0.36% |
| 184 | 4 |  |  |  | KB |  |  |  | Brd |  |  | uORF | PAS | 1.23% |
| 185 | 4 |  | GY |  | KB |  |  |  | Brd |  |  |  | PAS | 0.31% |
| 186 | 4 |  |  |  | KB | SXL-bs |  |  | Brd |  |  | uORF |  | 0.36% |
| 187 | 4 |  |  |  |  |  | Mos-PRE | UNR-bs |  |  | IRES | uORF |  | 0.51% |
| 188 | 4 |  | GY |  | KB |  |  |  |  |  | IRES | uORF |  | 0.41% |
| 189 | 4 | ADH_DRE |  |  |  | SXL-bs |  |  |  |  |  | uORF | PAS | 0.36% |
| 190 | 4 |  |  |  | KB | SXL-bs |  |  |  |  | IRES | uORF |  | 0.41% |
| 191 | 4 |  |  |  |  |  | Mos-PRE |  | Brd |  |  | uORF | PAS | 0.36% |
| 192 | 4 |  |  |  |  | SXL-bs |  |  | Brd |  |  | uORF | PAS | 0.82% |
| 193 | 4 |  |  |  |  | SXL-bs |  |  |  | CPE |  | uORF | PAS | 0.36% |
| 194 | 4 |  |  |  |  |  | Mos-PRE |  |  |  | IRES | uORF | PAS | 0.92% |
| 195 | 4 |  |  |  | KB | SXL-bs |  | UNR-bs |  |  |  | uORF |  | 0.26% |
| 196 | 4 |  |  |  |  |  |  | UNR-bs |  |  | IRES | uORF | PAS | 1.08% |
| 197 | 5 |  | GY |  |  | SXL-bs |  |  | Brd |  |  | uORF | PAS | 0.2% |
| 198 | 5 |  | GY |  |  | SXL-bs |  |  |  |  | IRES | uORF | PAS | 0.2% |
| 199 | 5 |  |  |  | KB |  | Mos-PRE | UNR-bs |  |  |  | uORF | PAS | 0.2% |
| 200 | 5 |  |  |  | KB | SXL-bs |  |  | Brd |  |  | uORF | PAS | 0.31% |
| 201 | 5 |  | GY |  |  |  |  | UNR-bs | Brd |  |  | uORF | PAS | 0.26% |
| 202 | 5 |  |  |  |  |  | Mos-PRE |  | Brd |  | IRES | uORF | PAS | 0.26% |
| 203 | 5 |  |  |  | KB | SXL-bs | Mos-PRE |  |  |  |  | uORF | PAS | 0.2% |
| 204 | 5 |  |  |  | KB |  |  |  | Brd |  | IRES | uORF | PAS | 0.51% |
| 205 | 5 |  |  |  |  |  | Mos-PRE | UNR-bs |  |  | IRES | uORF | PAS | 0.51% |
| 206 | 5 |  | GY |  | KB |  |  |  |  |  | IRES | uORF | PAS | 0.31% |
| 207 | 5 |  |  |  | KB |  |  | UNR-bs | Brd |  |  | uORF | PAS | 0.26% |
| 208 | 5 |  |  |  |  | SXL-bs |  |  | Brd |  | IRES | uORF | PAS | 0.46% |
| 209 | 5 |  |  |  | KB | SXL-bs |  |  |  |  | IRES | uORF | PAS | 0.36% |
| 210 | 5 |  | GY |  | KB |  |  |  | Brd |  |  | uORF | PAS | 0.31% |
| 211 | 5 |  |  |  | KB | SXL-bs |  | UNR-bs |  |  |  | uORF | PAS | 0.2% |
